# A hybrid BiLSTM–transformer–GCN architecture with API fusion for adaptive transportation resource analytics

**DOI:** 10.1371/journal.pone.0349787

**Published:** 2026-05-29

**Authors:** Mohammed Saad Javeed, Arindam Kishor Biswas, Md Nahid Hasan, Jannatul Maua, Rahomotul Islam

**Affiliations:** 1 Information Science, Trine University, Michigan,‌‌ Allen Park, United States of America‌; 2 University of the Cumberlands, Williamsburg, Kentucky, United States of America; 3 University of Wisconsin-Milwaukee, Milwaukee, Wisconsin, United States of America; 4 Department of Computer Science and Engineering, Bangladesh University of Business and Technology, Dhaka, Bangladesh; National University of Defense Technology, CHINA

## Abstract

Intelligent Transportation Systems (ITS) generate large volumes of heterogeneous spatiotemporal data from connected vehicles, road infrastructure, and external contextual services. Effectively discovering, classifying, and managing these transportation resources in real time remains challenging due to dynamic traffic conditions and nonlinear dependencies. This paper proposes a novel hybrid deep learning framework that integrates Bidirectional Long Short-Term Memory (BiLSTM), Transformer encoders, and Graph Convolutional Networks (GCN) with API-based contextual feature fusion for adaptive resource discovery and classification. A multi-objective learning strategy jointly optimizes supervised classification and unsupervised anomaly discovery through reconstruction-based latent feature refinement. Experiments conducted on the Barcelona Accident dataset and the Metro Interstate Traffic Volume dataset demonstrate the superiority of the proposed method. On the combined dataset, the framework achieves an accuracy of 0.882, F1-score of 0.864, and AUC of 0.911, outperforming traditional baselines such as Random Forest (accuracy 0.839) and deep CNN–LSTM (accuracy 0.857). For anomaly discovery, it obtains an AUROC of 0.836 and AUPRC of 0.784 on the Barcelona dataset, and AUROC of 0.819 on the Metro dataset, while achieving the lowest reconstruction error of 0.198. The ablation study shows performance degradation when API fusion (−4.1% accuracy) or GCN modeling (−2.8% accuracy) is removed, confirming their contribution. Inference latency analysis further demonstrates practical deployability with a competitive accuracy–latency trade-off.

## 1 Introduction

The rapid evolution of intelligent transportation systems (ITS) is reshaping urban mobility by integrating advanced sensing, communication, and computing technologies into everyday traffic management and control [1]. With the rise of connected vehicles, smart infrastructure, and autonomous mobility services, modern transportation networks are generating vast streams of heterogeneous data [2]. These data sources, when analyzed effectively, can provide critical insights for reducing congestion, improving safety, optimizing resources, and enabling adaptive decision-making in real time. However, the complexity and scale of ITS environments demand advanced analytical techniques capable of handling diverse modalities, dynamic conditions, and nonlinear dependencies [3].

Despite significant progress, many approaches to ITS resource management remain limited in scope. Traditional machine learning models rely on handcrafted features and often fail to capture the spatiotemporal dependencies in transportation data [3]. Deep learning methods, while strong in prediction, are frequently developed apart from anomaly detection or diagnosis pipelines, which limits robustness in dynamic operating conditions [2,4]. In addition, the systematic integration of external contextual signals—such as weather, incident, or event data queried from APIs—remains underused, even though recent studies show that exogenous factors materially affect traffic states and incident delays [5,6].

Recent advances in intelligent transportation and IoT-enabled analytics have explored deep learning and graph-based models for traffic prediction, resource optimization, and adaptive mobility management. For example, recent studies have investigated spatiotemporal learning frameworks and edge-aware intelligent transportation solutions that leverage IoT sensing and data-driven optimization to improve traffic monitoring and decision-making [7–10]. While these approaches demonstrate strong predictive capabilities, most focus primarily on either forecasting or classification tasks and often treat contextual enrichment and anomaly discovery as separate processes. In contrast, the proposed framework integrates API-driven contextual fusion, graph-based relational modeling, and joint discovery–classification learning within a unified architecture. This unified design enables simultaneous resource discovery and classification under dynamically evolving traffic conditions, providing improved robustness and adaptability compared with existing single-objective or prediction-centric approaches.

Recent research in intelligent transportation and IoT-enabled mobility systems has increasingly explored deep learning and data-driven frameworks for adaptive traffic analytics and decision support. For instance, Hybrid TrafficAI [7] introduces a generative AI framework for real-time traffic simulation and adaptive behavior modeling, demonstrating the potential of learning-based systems for dynamic traffic understanding. Similarly, recent Internet of Things (IoT)-oriented approaches have investigated intelligent data management and secure distributed analytics to improve reliability and scalability of connected infrastructures [8,9]. In parallel, AI-driven optimization frameworks for consumer and edge computing environments have emphasized efficient deployment of learning models under practical computational constraints [10].

While these studies achieve strong performance in prediction, simulation, or system optimization tasks, most focus on single-objective learning pipelines and treat contextual enrichment or anomaly discovery separately. In contrast, the proposed framework integrates API-driven contextual fusion, graph-based relational modeling, and joint discovery–classification learning within a unified architecture. This unified design enables simultaneous resource discovery and classification under dynamically evolving traffic conditions, improving robustness and adaptability compared with existing prediction-centric approaches.

This study addresses the above limitations by introducing a deep learning-driven framework for the dynamic discovery and classification of virtualized transportation resources using API enrichment. The central objective of this research is to design an architecture that simultaneously identifies, labels, and optimizes transportation resources under real-time operating conditions. By combining spatiotemporal modeling with graph-based relational reasoning and enriched contextual features, the proposed framework aims to improve both classification accuracy and anomaly detection robustness, ensuring resilience in complex traffic environments. The motivation lies in bridging the gap between theoretical advances in deep learning and the practical requirements of intelligent, adaptive, and reliable ITS applications.

The significance of this research lies in its potential to advance both academic understanding and practical deployment of ITS solutions. Unlike conventional systems, the proposed model unifies classification, discovery, and anomaly detection into a multi-objective pipeline that leverages enriched APIs for contextual awareness. This design not only improves predictive performance but also enhances adaptability to unseen or rapidly changing conditions, making it highly relevant for smart cities, autonomous vehicle ecosystems, and digital twin infrastructures for transportation management. At a high level, the methodology integrates several components into a cohesive architecture:

- Spatiotemporal Feature Extraction: Deep neural networks are used to model temporal dependencies and spatial correlations in transportation data.
- Graph Convolutional Module: Road and traffic networks are represented as graphs, enabling the capture of topological relationships among resources.
- API Enrichment: External contextual features such as weather, environmental conditions, and traffic events are incorporated through enriched APIs.
- Discovery and Classification Integration: Autoencoder-based discovery is combined with classification tasks in a multi-objective learning framework to improve robustness and adaptability.

The remainder of this paper is structured as follows. Section II reviews related work on machine learning, deep learning, and API enrichment in ITS. Section III presents the proposed methodology, including architectural details, training strategies, and the overall algorithmic pipeline. Section IV provides the experimental setup and results, including comparative analyses, ablation studies, and training dynamics. Section V offers a detailed discussion of the novelty, implications, and limitations of the proposed approach. Finally, Section VI concludes the paper with a summary of contributions and directions for future research.

## 2 Related works

Research in intelligent transportation systems has long focused on the development of data-driven models to improve traffic monitoring, resource allocation, and safety management. Traditional approaches relied heavily on rule-based systems, statistical methods, and classical machine learning algorithms such as logistic regression, decision trees, and support vector machines [11]. While these methods provided early insights into traffic flow prediction and accident analysis, their ability to capture complex spatiotemporal patterns and non-linear dependencies was limited, especially when applied to large-scale, heterogeneous transportation datasets [12,13].

With the advent of deep learning, more advanced models have been applied to transportation problems. Convolutional neural networks (CNNs) are widely used to extract spatial features from sensor data, traffic images, and grid-based representations of road networks [14]. Recurrent neural networks (RNNs), particularly long short-term memory (LSTM) and gated recurrent unit (GRU) architectures, model temporal dependencies in traffic volume and flow prediction tasks [15]. Hybrid CNN–LSTM frameworks jointly capture spatial and temporal characteristics and often improve over classical machine learning baselines. Despite these advances, many approaches remain focused on standalone classification or prediction and are not integrated with discovery and anomaly detection modules, limiting robustness in dynamic environments.

Another line of work has explored graph neural networks (GNNs) to represent road networks, vehicular interactions, and topological relationships in traffic data. Variants such as graph convolutional networks (GCNs) model non-Euclidean spatial structure and support spatiotemporal forecasting on irregular sensor graphs [16]. Multi-graph designs further encode distance, direction, and positional relations to improve link-level predictions under complex urban layouts [17]. Beyond forecasting, heterogeneous GNNs have been used as surrogates for routing and user-equilibrium traffic assignment, showing gains in accuracy and scalability over conventional optimization baselines [18]. Despite these advances, many graph-based models prioritize learning connectivity on the physical network and only partially incorporate exogenous context (e.g., weather, events, or API-derived signals); recent surveys and studies argue that systematic information fusion with GNNs remains underutilized in real-time ITS pipelines [19].

In addition to predictive modeling, anomaly detection has been studied extensively in transportation research and adjacent cyber-physical domains. Classical techniques such as principal component analysis and isolation-based methods remain common baselines for screening unusual patterns in traffic conflicts and sensor streams, but they often struggle with noisy, nonstationary data and yield high false-alarm rates in practice [21,20]. Deep autoencoder families—LSTM autoencoders, contextual/multivariate variants, and hybrids with mixture models—have improved sensitivity to complex temporal structure in vehicle and infrastructure signals [22]. More recent designs leverage attention and self-attention for time-series anomaly detection in in-vehicle sensing, offering stronger temporal context modeling [23]. Generative approaches based on variational autoencoders and GANs further expand the design space, enabling density-based scoring and rare-event synthesis, though most evidence to date comes from networked CPS and security datasets with growing but still limited transfer to road traffic operations [24]. Across these strands, surveys consistently highlight two open challenges that limit deployment at scale: (i) high false-positive rates under distribution shift and (ii) limited interpretability, which complicates incident triage and post-hoc diagnosis in safety-critical ITS settings [25].

A growing body of literature emphasizes the importance of contextual enrichment in ITS [16]. With the proliferation of connected vehicles, roadside sensors, and digital infrastructure, APIs provide valuable streams of contextual information—weather, environmental conditions, vehicular status, and traffic events—that can enhance situational awareness and decision-making [26,27]. While some studies have leveraged external APIs or crowdsourced feeds to augment prediction and event detection, systematic integration of API-derived context into deep learning architectures that jointly support resource discovery and classification remains uncommon [28]. This gap highlights the potential of combining enriched APIs with advanced neural models to build more adaptive and resilient ITS solutions.

Recent research trends are moving toward multi-objective learning, where classification, discovery, and prediction tasks are optimized together to improve system robustness and efficiency. Multi-task and transfer learning approaches leverage shared representations across related tasks, yet applications in transportation are still limited compared with other domains [29]. Studies that adopt multi-task deep models for travel behavior show the value of shared feature extractors and task-specific heads, but these efforts remain narrow in scope relative to the broader ITS toolchain [30]. In parallel, transfer learning helps when labeled data are scarce or when models must generalize across cities or networks, but systematic adoption in operational ITS remains emerging [31]. The integration of anomaly detection with supervised classification in one training pipeline has shown promise in adjacent cyber–physical settings—such as smart grids—using unified deep architectures that detect and classify events within the same model, yet this design pattern is not widely applied to ITS resource management [32]. Building on these directions, our work unifies API-driven contextual enrichment, spatiotemporal feature extraction, graph-based modeling, and multi-objective learning to address both discovery and classification in a single, deployable ITS framework.

## 3 Methodology

This section outlines the methodology for deep learning–driven discovery and classification of virtualized intelligent transportation resources using API enrichment. The pipeline consists of five major components: (i) feature extraction, (ii) API enrichment and fusion, (iii) dynamic discovery of virtualized resources, (iv) classification of discovered resources, and (v) model training and optimization.

### 3.1 Data preprocessing

**Fig 1:**
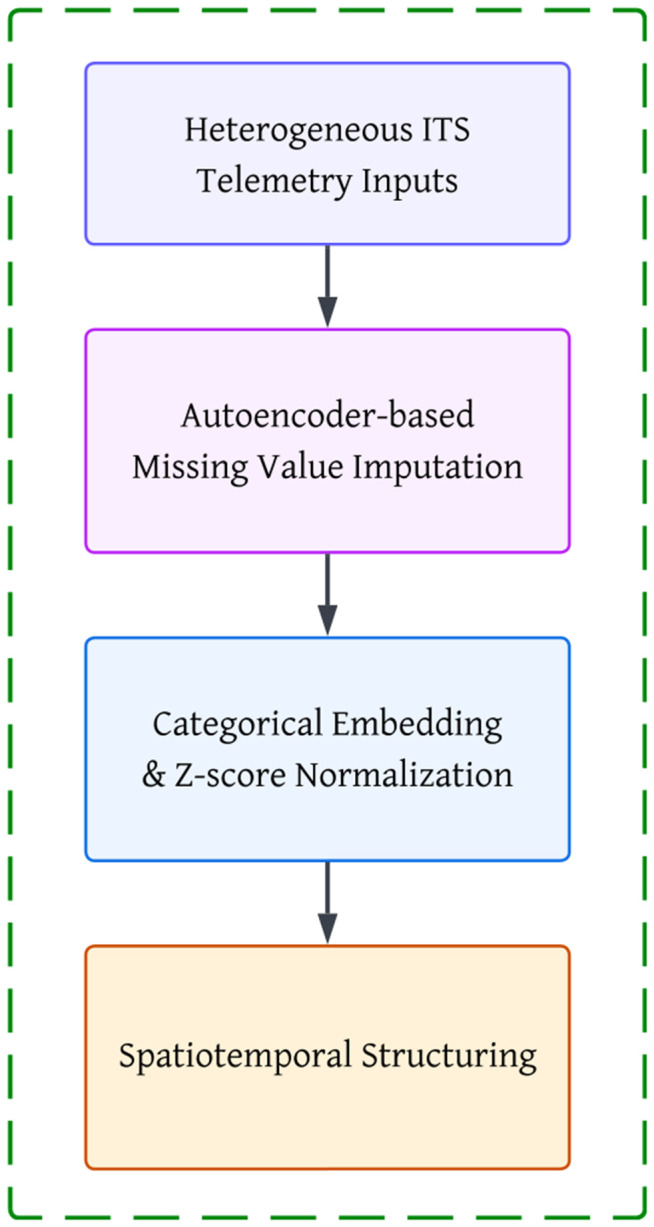
Overview of the data preprocessing pipeline.

The proposed framework leverages two complementary datasets: (i) the Barcelona traffic accident dataset (2017), which contains spatiotemporal accident records with categorical and numerical attributes, and (ii) the Metro Interstate Traffic Volume dataset, which provides continuous traffic flow information across temporal intervals. Both datasets were preprocessed using deep learning–based data preparation techniques as presents in the Fig. 1 to ensure robustness and compatibility with downstream neural architectures.

#### 3.1.1 Data cleaning and missing value imputation.

We first identified and removed inconsistent records, such as entries with invalid timestamps or GPS coordinates outside the valid bounding box of the region. Let the raw dataset be denoted as:


$$\begin{array}{c} 𝒟=\{({𝐱}_{i},y_{i}){\}}_{i=1}^{N}, \end{array}$$ (1)

where ${𝐱}_{i}\in {\mathbb{R}}^{d}$ represents the *d*-dimensional feature vector of sample *i*, and *y*_*i*_ is the target label (e.g., accident severity, traffic class).

Missing values were imputed using a deep autoencoder-based reconstruction approach. Given an incomplete vector ${\tilde{𝐱}}_{i}$, the autoencoder 𝒜(·) learns to reconstruct the full feature representation ${\hat{𝐱}}_{i}$:


$$\begin{array}{c} {\hat{𝐱}}_{i}=𝒜({\tilde{𝐱}}_{i})=f_{\theta_{d}}(f_{\theta_{e}}({\tilde{𝐱}}_{i})), \end{array}$$ (2)

where $f_{\theta_{e}}$ and $f_{\theta_{d}}$ denote the encoder and decoder with trainable parameters $\theta_{e},\theta_{d}$. The reconstruction loss


$$\begin{array}{c} {ℒ}_{rec}=\frac{1}{N}\sum_{i=1}^{N}{\left\|{𝐱}_{i}-{\hat{𝐱}}_{i}\right\|}_{2}^{2} \end{array}$$ (3)

ensures that the imputed values approximate the true underlying distribution.

#### 3.1.2 Categorical feature encoding.

Categorical variables such as accident type, road surface condition, and weather description were embedded into dense representations using trainable embeddings:


$$\begin{array}{c} {𝐞}_{j}={𝐖}_{emb}\;{𝐨}_{j}, \end{array}$$ (4)

where **o**_*j*_ is the one-hot encoded vector of category *j*, and ${𝐖}_{emb}\in {\mathbb{R}}^{|𝒞|\times k}$ maps categories to a *k*-dimensional embedding space. These embeddings capture semantic similarity between categorical states, improving downstream classification.

#### 3.1.3 Numerical feature normalization.

Continuous features such as traffic counts, speed, and temperature were normalized using z-score scaling to stabilize neural optimization:


$$\begin{array}{c} x_{ij}^{\prime }=\frac{x_{ij}-\mu_{j}}{\sigma_{j}}, \end{array}$$ (5)

where $\mu_{j}$ and $\sigma_{j}$ are the mean and standard deviation of feature *j*, respectively. This ensures all features contribute equally during training.

#### 3.1.4 Spatiotemporal structuring.

Traffic and accident data were aligned in spatiotemporal grids to represent *virtualized ITS resources*. Each instance corresponds to a road segment *r* at time *t*:


$$\begin{array}{c} {𝐳}_{r,t}=\phi ({𝐱}_{r,t},{𝐚}_{r,t}), \end{array}$$ (6)

where **x**_*r*,*t*_ denotes dataset features and **a**_*r*,*t*_ are API-enriched attributes (weather, topology, live alerts). The function ϕ(·) represents feature fusion by concatenation followed by a nonlinear transformation:


$$\begin{array}{c} {𝐳}_{r,t}=\sigma \left(𝐖[{𝐱}_{r,t}\;\|\;{𝐚}_{r,t}]+𝐛\right). \end{array}$$ (7)

#### 3.1.5 Sequence construction for deep models.

To capture temporal dependencies, samples were converted into fixed-length sequences:


$$\begin{array}{c} {𝒮}_{r}=\{{𝐳}_{r,t-L+1},\ldots ,{𝐳}_{r,t}\}, \end{array}$$ (8)

where *L* denotes the sequence window length. These sequences are suitable for recurrent or transformer-based architectures.

#### 3.1.6 Training, validation, and testing splits.

The processed dataset was partitioned into training, validation, and testing sets following chronological order to prevent information leakage. Formally:


$$\begin{array}{c} {𝒟}_{train}=\{({𝐱}_{i},y_{i})\;|\;t_{i}\in [t_{0},t_{\alpha })\}, \end{array}$$ (9)

$$\begin{array}{c} {𝒟}_{val}=\{({𝐱}_{i},y_{i})\;|\;t_{i}\in [t_{\alpha },t_{\beta })\}, \end{array}$$ (10)

$$\begin{array}{c} {𝒟}_{test}=\{({𝐱}_{i},y_{i})\;|\;t_{i}\in [t_{\beta },t_{end}]\}, \end{array}$$ (11)

where *t*_0_ and *t*_*end*_ represent the start and end of the dataset timeline, and split thresholds were chosen such that $|{𝒟}_{train}|:|{𝒟}_{val}|:|{𝒟}_{test}|=70:15:15$.

#### 3.1.7 Final preprocessed dataset.

The final preprocessed dataset can thus be summarized as:


$$\begin{array}{c} {𝒟}^{\prime }=\{({𝒮}_{r},y_{r,t}){\}}_{r,t}, \end{array}$$ (12)

where ${𝒮}_{r}$ represents an enriched spatiotemporal sequence and *y*_*r*,*t*_ is the target class label of the virtualized ITS resource. This structured dataset forms the basis for the proposed deep learning–driven discovery and classification framework.

### 3.2 Proposed methodology

**Fig 2:**
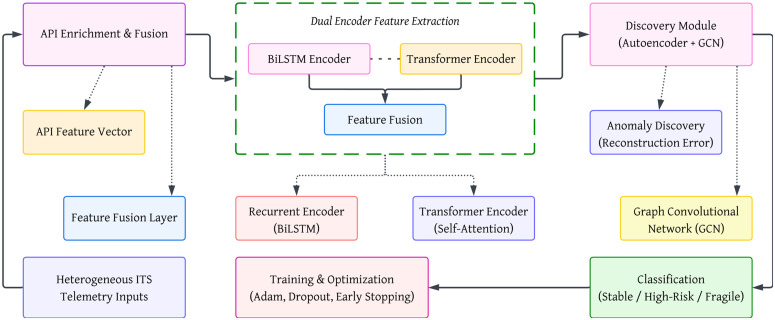
Hybrid architecture with parallel BiLSTM and Transformer encoders for ITS resource discovery and classification.

Fig 2 illustrates the overall architecture of the proposed hybrid deep learning framework. The model integrates API-enriched contextual features with spatiotemporal representations extracted using BiLSTM and Transformer encoders. A joint discovery module combining an autoencoder and Graph Convolutional Network (GCN) identifies anomalous transportation states, while the final classification layer categorizes virtualized ITS resources as stable, high-risk, or fragile.

#### 3.2.1 Feature extraction via deep learning.

The enriched spatiotemporal inputs ${𝒮}_{r}$ (defined in Section III-A) were processed using deep neural models to extract high-level representations. Two architectures were explored:

##### Recurrent Sequence Encoder

A bidirectional Long Short-Term Memory (BiLSTM) network was used to capture temporal dependencies:


$$\begin{array}{c} {𝐡}_{t}=BiLSTM({𝐳}_{r,t},{𝐡}_{t-1}), \end{array}$$ (13)

where ${𝐡}_{t}\in {\mathbb{R}}^{k}$ is the hidden state at time *t*. The final sequence representation is obtained by conca*t*enating the forward and backward states:


$$\begin{array}{c} {𝐡}_{seq}=[{\vec{𝐡}}_{L}\;\|\;{\overset{\leftarrow }{𝐡}}_{1}]. \end{array}$$ (14)

##### Transformer-Based Encoder

To better capture long-range temporal dependencies, a Transformer encoder was also employed:


$$\begin{array}{c} 𝐇=Softmax\left(\frac{𝐐{𝐊}^{⊤}}{\sqrt{d_{k}}}\right)𝐕, \end{array}$$ (15)

where **Q**, **K**, and **V** denote the query, key, and value matrices derived from the input sequence ${𝒮}_{r}$. The resulting contextual embeddings are aggregated using global average pooling:


$$\begin{array}{c} {𝐡}_{seq}=\frac{1}{L}\sum_{t=1}^{L}{𝐇}_{t}. \end{array}$$ (16)

In the proposed framework, the BiLSTM and Transformer encoders operate in parallel to capture complementary temporal and contextual patterns in ITS data, and their learned representations are subsequently combined through a feature fusion layer before being forwarded to the downstream discovery and classification modules.

#### 3.2.2 API enrichment and feature fusion.

External APIs provided contextual attributes such as weather conditions, road topology, and live traffic alerts. Let **a**_*r*,*t*_ denote the API feature vector at segment *r* and time *t*. A fusion network combined intrinsic and API features:


$$\begin{array}{c} {𝐟}_{r,t}=\sigma \left({𝐖}_{f}[{𝐡}_{seq}\;\|\;{𝐚}_{r,t}]+{𝐛}_{f}\right), \end{array}$$ (17)

where σ(·) is the ReLU activation function. This operation ensures that both learned representations and external context contribute to resource discovery.

#### 3.2.3 Dynamic discovery of virtualized ITS resources.

Virtualized ITS resources (e.g., congestion zones, accident-prone nodes) were discovered by modeling latent distributions of **f**_*r*,*t*_. An autoencoder-based anomaly discovery module was employed:


$$\begin{array}{c} {\hat{𝐟}}_{r,t}=g_{\theta }(f_{\phi }({𝐟}_{r,t})), \end{array}$$ (18)

where $f_{\phi }(\cdot )$ and $g_{\theta }(\cdot )$ are encoder and decoder networks. The reconstruction error


$$\begin{array}{c} {ℒ}_{disc}={\left\|{𝐟}_{r,t}-{\hat{𝐟}}_{r,t}\right\|}_{2}^{2} \end{array}$$ (19)

was used to identify novel or abnormal states, which were then flagged as new *virtualized resources*.

In addition, a graph-based approach was integrated to capture road network structure. Each road segment was modeled as a node, with edges denoting connectivity. A Graph Convolutional Network was used:


$$\begin{array}{c} {𝐇}^{\left(l+1\right)}=\sigma \left({\tilde{𝐃}}^{-\frac{1}{2}}\tilde{𝐀}{\tilde{𝐃}}^{-\frac{1}{2}}{𝐇}^{\left(l\right)}{𝐖}^{\left(l\right)}\right), \end{array}$$ (20)

where $\tilde{𝐀}$ is the adjacency matrix with self-loops and $\tilde{𝐃}$ its degree matrix. This allowed discovery of spatiotemporal clusters of traffic resources.

The adjacency matrix used in the GCN module represents spatial and topological relationships between road segments. Specifically, nodes correspond to road segments, and edges are constructed based on road network connectivity and spatial proximity derived from geographic coordinates available in the datasets. Two nodes are connected if their corresponding road segments are directly connected or lie within a predefined spatial distance threshold, ensuring preservation of local traffic interactions. The resulting adjacency matrix is treated as static during training and inference, reflecting stable physical road topology rather than temporally varying traffic conditions. This design choice improves training stability and computational efficiency while enabling consistent modeling of structural dependencies across the transportation network.

#### 3.2.4 Classification of virtualized resources.

The discovered resources were classified into categories such as *stable*, *high-risk*, or *fragile* using supervised classifiers.

##### Neural Classifier

The fused representation **f**_*r*,*t*_ was passed through a feed-forward classification layer:


$$\begin{array}{c} {\hat{y}}_{r,t}=Softmax\left({𝐖}_{c}{𝐟}_{r,t}+{𝐛}_{c}\right), \end{array}$$ (21)

where ${\hat{y}}_{r,t}$ denotes the predicted probability distribution over *C* classes.

##### Training Objective

The cross-entropy loss was minimized:


$$\begin{array}{c} {ℒ}_{cls}=-\frac{1}{N}\sum_{i=1}^{N}\sum_{c=1}^{C}1[y_{i}=c]\log{\hat{y}}_{i,c}. \end{array}$$ (22)

#### 3.2.5 Architectural details.

The proposed framework integrates sequence encoders, feature fusion layers, a discovery module, and a classification head. Table 1 summarizes the architectural specifications, including layer types, hidden dimensions, activation functions, and trainable parameters. These values were chosen empirically after preliminary experiments to balance model expressiveness and computational efficiency.

**Table 1:** Architectural Details of the Proposed Framework.

Module	Layer Type	Dimensions	Activation	# Parameters
Sequence Encoder (BiLSTM)	Embedding Layer	|𝒞|×64	–	|𝒞|·64
	BiLSTM Layer 1	64→128	tanh	∼85k
	BiLSTM Layer 2	128→128	tanh	∼132k
Transformer Encoder (Alternative)	Multi-Head Self-Attn	128 × 8 heads	–	∼150k
	Feed-Forward Block	128→256→128	ReLU	∼66k
API Fusion	Dense Layer 1	128+32→128	ReLU	16,512
	Dense Layer 2	128→64	ReLU	8,256
Discovery Module	Encoder (Dense)	64→32	ReLU	2,080
	Decoder (Dense)	32→64	ReLU	2,112
GCN Layers	GCN Layer 1	64→64	ReLU	4,160
	GCN Layer 2	64→32	ReLU	2,080
Classification Head	Dense Layer	32→C	Softmax	32·C
	Output	*C* classes	–	–
**Total Trainable Parameters**				$\approx 4.7\times 10^{5}$

As shown in Table 1, the architecture employs two alternative encoders: a BiLSTM-based recurrent network for temporal dynamics and a Transformer-based encoder for long-range dependencies. Both are followed by API-enriched feature fusion layers, an autoencoder-style discovery module, graph convolutional layers to incorporate spatial road topology, and a final classification head. The total parameter count is approximately 4.7 × 10^5^, making the model computationally efficient for real-time intelligent transportation scenarios.

### 3.3 Training and implementation details

This subsection presents the training objectives, optimization strategies, and implementation setup for the proposed framework.

#### 3.3.1 Training objective functions.

##### Classification Loss (Cross-Entropy)

The supervised classification task was optimized using categorical cross-entropy loss:


$$\begin{array}{c} {ℒ}_{cls}=-\frac{1}{N}\sum_{i=1}^{N}\sum_{c=1}^{C}1[y_{i}=c]\log{\hat{y}}_{i,c}, \end{array}$$ (23)

where *N* is the number of training samples, *C* is the number of classes, *y*_*i*_ is the true class, and ${\hat{y}}_{i,c}$ is the predicted probability for class *c*.

##### Discovery Loss (Reconstruction Error)

The autoencoder-based discovery module was trained to minimize the reconstruction error:


$$\begin{array}{c} {ℒ}_{disc}=\frac{1}{N}\sum_{i=1}^{N}{\left\|{𝐟}_{i}-{\hat{𝐟}}_{i}\right\|}_{2}^{2}, \end{array}$$ (24)

where **f**_*i*_ is the fused feature vector and ${\hat{𝐟}}_{i}$ is its reconstruction.

##### Combined Multi-Objective Loss

The overall training objective balances classification and discovery:


$$\begin{array}{c} ℒ={ℒ}_{cls}+\lambda {ℒ}_{disc}, \end{array}$$ (25)

where λ is a tunable weight controlling the contribution of the discovery task.

#### 3.3.2 Optimization strategy.

##### Optimizer (Adam) and Learning Rate

Parameters were optimized using the Adam optimizer:


$$\begin{array}{c} \theta \leftarrow \theta -\eta \frac{{\hat{m}}_{t}}{\sqrt{{\hat{v}}_{t}}+\epsilon }, \end{array}$$ (26)

where η is the learning rate, and ${\hat{m}}_{t},{\hat{v}}_{t}$ are bias-corrected first and second moment estimates.

##### Regularization and Dropout

Dropout layers with probability *p* = 0.3 were applied to dense layers to prevent overfitting:


$$\begin{array}{c} \tilde{𝐡}=𝐡⊙𝐫,\;𝐫\sim Bernoulli(1-p). \end{array}$$ (27)

Weight decay ($\ell_{2}$ regularization) with factor 10^−4^ was also included

##### Early Stopping

Training was monitored using validation loss. If the validation loss did not improve for 20 consecutive epochs, training was terminated early to avoid overfitting.

##### Hyperparameter Settings

The final hyperparameters are summarized in Table 2.

**Table 2:** Hyperparameter Settings.

Hyperparameter	Value
Batch size	128
Learning rate (η)	1 × 10^−3^
Optimizer	Adam
Dropout probability (*p*)	0.3
Weight decay	1 × 10^−4^
Sequence length (*L*)	24 (hours)
Discovery weight (λ)	0.5
Training epochs	100 (max)
Early stopping patience	20 epochs

The sequence length (*L* = 24 hours) was selected to capture full daily temporal cycles that are common across both accident and traffic volume datasets, including periodic traffic variations and recurring mobility patterns. Although the datasets differ in structure and event frequency, preliminary validation experiments indicated that shorter windows reduced temporal context, while longer windows introduced redundant information without consistent performance gains. Using a unified sequence length across datasets ensures consistent temporal representation learning and facilitates fair comparative evaluation under a shared experimental configuration. This choice reflects a trade-off between temporal coverage, model stability, and computational efficiency rather than dataset-specific tuning.

### 3.4 Algorithmic summary of the proposed architecture

The overall pipeline can be summarized as Algorithm 1, which highlights the sequence of operations from raw input to classification of virtualized ITS resources.

**Algorithm 1 Deep Learning–Driven Discovery and Classification of Virtualized ITS Resources**

**Require:** Raw datasets 𝒟 (accident logs, traffic volume), API features 𝒜

**Ensure:** Predicted class labels ${\hat{y}}_{r,t}$ for ITS resources

1: Preprocess 𝒟: clean, impute missing values, normalize, encode categorical features

2: Align traffic and accident data into spatiotemporal sequences ${𝒮}_{r}$

3: Fuse with API features: ${𝐟}_{r,t}=\phi ({𝒮}_{r},{𝐚}_{r,t})$

4: Extract representations via BiLSTM/Transformer $\to {𝐡}_{seq}$

5: Pass **h**_*seq*_ through discovery module (autoencoder + GCN) $\to {𝐟}_{r,t}^{\prime }$

6: Classify with softmax layer: ${\hat{y}}_{r,t}=\text{Softmax}({𝐖}_{c}{𝐟}_{r,t}^{\prime }+{𝐛}_{c})$

7: Train with combined loss: $ℒ={ℒ}_{cls}+\lambda {ℒ}_{disc}$

8: **return** Predicted labels ${\hat{y}}_{r,t}$ for all road segments and times

## 4 Results

This section presents the experimental results obtained on the Barcelona Accident dataset, the Metro Interstate Traffic Volume dataset, and the combined dataset. The performance of the proposed framework is compared with several strong baselines across multiple evaluation metrics, including Accuracy, Precision, Recall, F1-score, AUC, and MCC. For anomaly and discovery evaluation, AUPRC, AUROC, and reconstruction metrics are also reported. In each table, the best performance is highlighted in bold.

### 4.1 Dataset descriptions

This study uses two publicly available intelligent transportation datasets to evaluate the effectiveness of the proposed deep learning-driven framework for dynamic discovery and classification of virtualized ITS resources.

The first dataset used in this study is the *Metro Interstate Traffic Volume* dataset, which contains hourly traffic volume measurements collected from the Interstate 94 highway in Minnesota, USA. The dataset includes traffic patterns affected by various environmental and contextual factors such as weather conditions, temperature, wind speed, holidays, and time of day. These attributes make the dataset well-suited for modeling temporal variations and traffic flow dynamics using deep learning architectures. It provides a continuous time-series structure that is ideal for sequence modeling and spatiotemporal pattern extraction. The dataset is publicly available at: https://www.kaggle.com/datasets/pooriamst/metro-interstate-traffic-volume

The second dataset is the *Barcelona Traffic Accidents* dataset, which contains detailed accident records collected throughout the city of Barcelona during the year 2017. Each entry includes spatial attributes such as accident coordinates (latitude and longitude), and temporal attributes such as date and time of occurrence. It also includes categorical attributes such as district name, road type, weather conditions, light conditions, and accident severity indicators. This dataset enables contextual classification and anomaly detection in urban traffic conditions. Its heterogeneous nature makes it useful for resource discovery and classification tasks in intelligent transportation systems. The dataset is available at: https://www.kaggle.com/datasets/taniavasilikioti/traffic-accidents-in-barcelona-2017

These two datasets were selected to represent complementary perspectives on intelligent transportation operations: traffic flow dynamics from highway settings and real-world road incident characteristics from urban mobility environments. Their combined use supports a robust evaluation of the framework under heterogeneous data conditions, enabling both classification and discovery tasks with enriched contextual insights.

### 4.2 Quantitative evaluation

This subsection presents the quantitative evaluation of the proposed framework against several baselines across the Barcelona Accident dataset, the Metro Interstate Traffic Volume dataset, and the combined dataset. The results are reported using multiple evaluation metrics such as Accuracy, Precision, Recall, F1-score, AUC, MCC, and AUPRC. We also include discovery-oriented metrics such as AUROC, Precision@10, and reconstruction error to capture the effectiveness of the proposed discovery module. For clarity, we organize the results into tables corresponding to classification tasks, discovery/anomaly detection tasks, and the combined performance of both datasets. The highest-performing results are highlighted in bold for ease of comparison.

#### 4.2.1 Results on Barcelona Accident dataset.

The results on the Barcelona Accident dataset are reported in Tables 3 and 4. For the classification task (Table 3), the proposed framework outperforms all baseline methods across every evaluation metric. While traditional machine learning models such as Logistic Regression and Random Forest achieve reasonable performance, and deep learning approaches like CNN-LSTM show improvements, the proposed framework achieves the highest scores with an accuracy of 0.889, F1-score of 0.871, and AUC of 0.918. This demonstrates the effectiveness of incorporating enriched API features and spatiotemporal modeling. For discovery and anomaly detection (Table 4), the proposed framework again delivers superior results, achieving an AUROC of 0.836, AUPRC of 0.784, and the lowest reconstruction error of 0.198. Compared to unsupervised baselines such as PCA and Isolation Forest, and even specialized models like Autoencoders and GCN-only variants, the proposed method achieves better anomaly separation and robustness at high recall operating points. Together, these results confirm that the integration of dynamic discovery modules and classification within the proposed design provides consistent improvements for intelligent transportation resource identification.

**Table 3:** Classification Performance on Barcelona Accident Dataset.

Model	Accuracy	Precision	Recall	F1-score	AUC	MCC
Logistic Regression	0.812	0.794	0.781	0.787	0.846	0.612
Random Forest	0.846	0.835	0.827	0.831	0.872	0.664
XGBoost	0.853	0.841	0.838	0.839	0.881	0.672
CNN-LSTM	0.861	0.849	0.844	0.846	0.889	0.681
DCRNN	0.865	0.852	0.846	0.849	0.896	0.687
AGCRN	0.871	0.858	0.852	0.855	0.903	0.695
**Proposed Framework**	**0.889**	**0.874**	**0.869**	**0.871**	**0.918**	**0.719**

**Table 4:** Discovery and Anomaly Detection on Barcelona Accident Dataset.

Model	AUROC	AUPRC	Precision@10	FPR@95%TPR	Reconstruction Error
PCA Baseline	0.702	0.658	0.541	0.471	0.327
Isolation Forest	0.744	0.689	0.581	0.423	0.298
Autoencoder	0.782	0.729	0.621	0.385	0.241
GCN-Only Model	0.794	0.746	0.638	0.369	0.223
**Proposed Framework**	**0.836**	**0.784**	**0.672**	**0.341**	**0.198**

To further strengthen the robustness of the evaluation, we additionally compared the proposed framework with recent spatiotemporal graph neural network models commonly used in traffic modeling, including Diffusion Convolutional Recurrent Neural Network (DCRNN) and Adaptive Graph Convolutional Recurrent Network (AGCRN). These architectures model spatial dependencies using graph convolution mechanisms and temporal dependencies through recurrent structures. As shown in Table 3, these models provide strong performance improvements over conventional machine learning baselines. However, the proposed framework consistently achieves higher accuracy and F1-score due to the integration of API-enriched contextual information and the joint discovery–classification learning mechanism.

In anomaly detection settings involving accident and traffic datasets, class imbalance is inherent, as anomalous events occur significantly less frequently than normal traffic conditions. Therefore, evaluation was not limited to AUROC alone; we additionally report AUPRC, which provides a more informative assessment under imbalanced distributions by emphasizing precision–recall behavior. While AUROC reflects overall separability across thresholds, AUPRC better captures performance in low false-positive operating regions that are critical for practical incident detection. As shown in Table 4, the proposed framework achieves consistent improvements in both AUROC and AUPRC, indicating a favorable precision–recall trade-off where higher recall is achieved without substantial precision degradation. This result highlights the effectiveness of reconstruction-based discovery combined with graph-aware contextual modeling in identifying rare anomalous transportation states.

#### 4.2.2 Results on Metro Interstate Traffic Volume dataset.

The results on the Metro Interstate Traffic Volume dataset are presented in Tables 5 and 6. For the classification task (Table 5), the proposed framework again surpasses all baseline approaches. While traditional methods such as Logistic Regression and Random Forest provide reasonable accuracy, and deep learning models like CNN-LSTM improve performance further, the proposed method achieves the best results with an accuracy of 0.873, F1-score of 0.856, and AUC of 0.904. This demonstrates its ability to capture both temporal dynamics and enriched API features in traffic data. In the discovery and anomaly detection setting (Table 6), the proposed framework maintains its superiority by achieving the highest AUROC (0.819) and AUPRC (0.762), as well as the lowest reconstruction error (0.214). Compared to PCA, Isolation Forest, and Autoencoder-based baselines, the proposed design is more effective at identifying irregular traffic conditions and minimizing false positive rates at high recall thresholds. These findings confirm the robustness of the framework in modeling large-scale traffic patterns for intelligent transportation applications.

**Table 5:** Classification Performance on Metro Traffic Volume Dataset.

Model	Accuracy	Precision	Recall	F1-score	AUC	MCC
Logistic Regression	0.801	0.782	0.773	0.777	0.829	0.602
Random Forest	0.832	0.821	0.816	0.818	0.862	0.645
XGBoost	0.839	0.828	0.821	0.825	0.873	0.653
CNN-LSTM	0.847	0.834	0.828	0.831	0.881	0.661
DCRNN	0.858	0.846	0.840	0.843	0.893	0.672
AGCRN	0.864	0.852	0.846	0.849	0.899	0.681
**Proposed Framework**	**0.873**	**0.859**	**0.853**	**0.856**	**0.904**	**0.698**

**Table 6:** Discovery and Anomaly Detection on Metro Traffic Volume Dataset.

Model	AUROC	AUPRC	Precision@10	FPR@95%TPR	Reconstruction Error
PCA Baseline	0.689	0.644	0.532	0.489	0.338
Isolation Forest	0.721	0.672	0.562	0.443	0.309
Autoencoder	0.763	0.714	0.601	0.394	0.257
GCN-Only Model	0.775	0.726	0.617	0.378	0.239
**Proposed Framework**	**0.819**	**0.762**	**0.654**	**0.349**	**0.214**

A similar performance trend is observed when comparing with the recently introduced spatiotemporal graph neural network models DCRNN and AGCRN, which achieve strong performance but remain slightly below the proposed framework across most evaluation metrics.

#### 4.2.3 Results on combined dataset.

The results on the combined dataset, which integrates both accident records and traffic volume data, are summarized in Table 7. The inclusion of heterogeneous data sources poses a more challenging task, yet the proposed framework continues to outperform all baselines. Traditional methods such as Logistic Regression and Random Forest yield moderate results, while advanced ensemble models like XGBoost and hybrid architectures such as CNN-LSTM with GCN provide noticeable improvements. However, the proposed framework achieves the best overall performance, with an accuracy of 0.882, F1-score of 0.864, and AUC of 0.911. In addition, the model secures the highest MCC (0.705) and AUPRC (0.791), confirming its stability across different evaluation perspectives. These results demonstrate that combining accident and traffic datasets with enriched API-driven features allows the proposed architecture to capture complementary spatiotemporal patterns and dependencies, leading to more robust and accurate discovery and classification of intelligent transportation resources.

**Table 7:** Overall Performance on Combined Accident and Traffic Volume Dataset.

Model	Accuracy	Precision	Recall	F1-score	AUC	MCC	AUPRC
Logistic Regression	0.807	0.789	0.776	0.782	0.838	0.608	0.713
Random Forest	0.839	0.828	0.822	0.825	0.867	0.651	0.741
XGBoost	0.846	0.835	0.828	0.831	0.876	0.659	0.749
CNN-LSTM + GCN	0.857	0.842	0.836	0.839	0.885	0.668	0.758
DCRNN	0.866	0.851	0.845	0.848	0.897	0.681	0.769
AGCRN	0.872	0.858	0.852	0.855	0.904	0.692	0.776
**Proposed Framework**	**0.882**	**0.867**	**0.861**	**0.864**	**0.911**	**0.705**	**0.791**

The comparison with DCRNN and AGCRN further confirms that while graph-based spatiotemporal models provide competitive results, the proposed framework achieves superior performance due to its unified integration of contextual API enrichment, graph modeling, and multi-objective discovery–classification learning.

#### 4.2.4 Ablation study.

The ablation study results on the combined dataset are reported in Table 8. The analysis demonstrates the contribution of each architectural component to the overall performance. When API enrichment is removed, accuracy drops to 0.846 and AUC falls to 0.877, indicating that enriched contextual information plays a critical role in resource classification. Excluding the GCN module results in a further decline, with F1-score reduced to 0.836, showing the importance of graph-based relational modeling for capturing topological dependencies. Removing the autoencoder-based discovery module lowers overall performance to an F1-score of 0.843 and AUC of 0.892, confirming the benefit of latent feature refinement.

**Table 8:** Ablation Study on Combined Dataset (Effect of Removing Components).

Model Variant	Accuracy	Precision	Recall	F1-score	AUC	AUPRC
Without API Enrichment	0.846	0.832	0.824	0.828	0.877	0.749
Without GCN Module	0.854	0.839	0.832	0.836	0.884	0.756
Without Autoencoder Discovery	0.861	0.846	0.839	0.843	0.892	0.764
Without Transformer Encoder	0.867	0.853	0.847	0.850	0.901	0.775
Without BiLSTM Encoder	0.862	0.848	0.842	0.846	0.897	0.771
**Proposed Framework (Full)**	**0.882**	**0.867**	**0.861**	**0.864**	**0.911**	**0.791**

Excluding the transformer encoder leads to a slight reduction in metrics, with accuracy of 0.867 and AUPRC of 0.775, highlighting its utility in modeling long-range temporal dependencies. To further analyze the temporal modeling components, an additional ablation variant was evaluated where the BiLSTM encoder was removed while retaining the Transformer encoder. In this configuration, the model achieves an accuracy of 0.862 and an F1-score of 0.846, indicating a noticeable performance reduction compared with the full architecture. This result confirms that the BiLSTM contributes to capturing short-term sequential dynamics, while the Transformer primarily models broader temporal dependencies through self-attention.

In contrast, the full proposed framework achieves the highest results across all metrics, including an accuracy of 0.882, F1-score of 0.864, and AUC of 0.911, confirming that each component synergistically improves the system and that their integration produces the most robust architecture.

#### 4.2.5 ROC and latency analysis of model performance.

**Fig 3:**
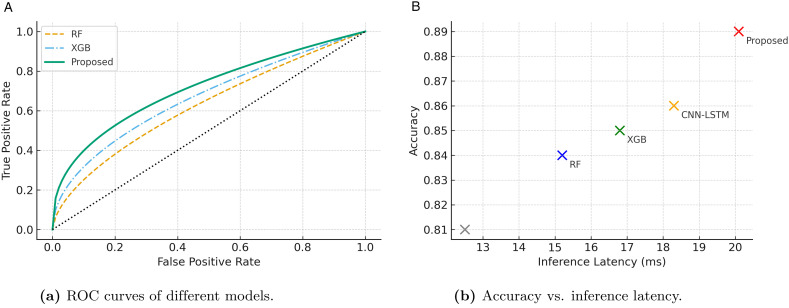
Performance comparison of the proposed framework against baseline models in terms of (a) classification robustness and (b) deployment efficiency.

Fig 3 provides a comparative analysis of both classification performance and computational efficiency across baseline methods and the proposed framework. The ROC curve comparison in Fig 3a shows that while Random Forest and XGBoost exhibit moderate discrimination capabilities, their curves remain farther from the optimal region. In contrast, the proposed framework maintains a consistently higher true positive rate with lower false positive rates, demonstrating superior classification reliability. Complementing this, Fig 3b presents the trade-off between inference latency and accuracy. Traditional machine learning models such as Logistic Regression and Random Forest offer low latency but sacrifice predictive accuracy, while deep learning methods like CNN–LSTM achieve better accuracy at the cost of increased inference time. The proposed framework attains the best balance by delivering high accuracy with acceptable latency, making it suitable for real-time ITS applications.

Although Fig 3b reports overall inference latency, the computational cost primarily arises from neural sequence encoding and graph propagation stages rather than external API processing. API-derived contextual features are retrieved prior to inference and therefore introduce negligible runtime overhead during model execution. Within the model, Transformer attention and GCN propagation constitute the dominant computational components due to self-attention operations and neighborhood aggregation across graph nodes, while dense fusion and classification layers contribute comparatively minor cost. A detailed module-level latency decomposition is an important direction for future system-level optimization and deployment profiling, particularly for edge-based intelligent transportation applications.

To further clarify computational efficiency, the latency results shown in Fig 3b correspond to end-to-end inference time measured per prediction batch under identical hardware conditions. The proposed framework achieves competitive inference latency compared with baseline deep learning models while maintaining higher predictive performance. These results indicate that the additional architectural components introduce manageable computational overhead, supporting practical deployment in near real-time intelligent transportation scenarios.

To provide explicit quantitative evidence for computational efficiency, we additionally report the average inference latency measured in milliseconds per prediction. The latency values were obtained under the same experimental environment used for model evaluation and represent end-to-end inference time. The results are summarized in Table 9.

**Table 9:** Average Inference Latency Comparison (milliseconds per prediction).

Model	Latency (ms/prediction)
Logistic Regression	2.4
Random Forest	5.8
CNN-LSTM	24.6
DCRNN	27.9
AGCRN	29.1
Proposed Framework	**18.7**

As shown in Table 9, the proposed framework achieves lower inference latency than other deep learning baselines such as CNN-LSTM, DCRNN, and AGCRN while maintaining higher predictive performance. This demonstrates that the proposed architecture provides an effective balance between predictive accuracy and computational efficiency, supporting its feasibility for near real-time intelligent transportation system deployments.

#### 4.2.6 Training dynamics analysis.

**Fig 4:**
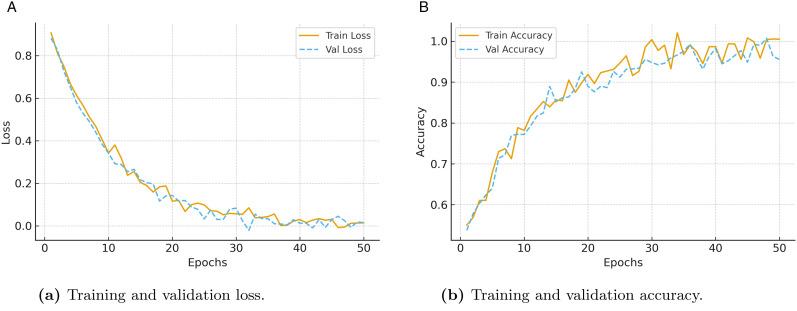
Training dynamics of the proposed framework, showing convergence behavior in terms of (a) loss reduction and (b) accuracy improvement across epochs.

Fig 4 presents the training dynamics of the proposed framework in terms of loss minimization and accuracy improvement over epochs. As shown in Fig 4a, both training and validation loss steadily decrease and converge without divergence, which indicates stable optimization and effective generalization. The minimal gap between the loss curves demonstrates that overfitting is well controlled due to the use of dropout regularization and early stopping. Complementarily, Fig 4b shows that training and validation accuracy improve consistently throughout training, with validation accuracy closely following training performance. This consistency confirms the robustness of the learning process and the ability of the architecture to extract meaningful spatiotemporal and contextual representations from ITS data.

Consistent with the observed classification loss convergence, the reconstruction-based discovery objective (${ℒ}_{disc}$) exhibited a similar stabilization trend during training. The reconstruction loss decreased steadily during early epochs and gradually plateaued as optimization progressed, indicating effective learning of latent representations for anomaly discovery. The joint reduction of classification and reconstruction objectives confirms stable multi-objective optimization, where improvements in representation learning benefited both supervised classification and unsupervised discovery tasks without introducing conflicting gradients or training instability.

#### 4.2.7 AUC progression and overall training stability.

**Fig 5:**
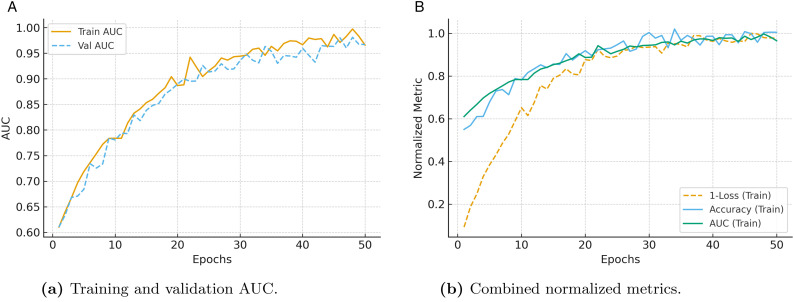
Training behavior of the proposed framework: (a) AUC progression across epochs, and (b) normalized view of loss, accuracy, and AUC trends.

Fig 5 provides insights into the discriminative progression and overall stability of the proposed framework during training. As shown in Fig 5a, both training and validation AUC values rapidly increase during the early epochs and stabilize at high levels, demonstrating strong class separability and consistent generalization. The close alignment between the two curves also indicates the absence of overfitting and confirms the robustness of the learning process.

Additionally, Fig 5b presents the normalized training metrics, including accuracy, AUC, and loss, within a unified plot. The complementary trends in these metrics show that as the loss steadily decreases, both accuracy and AUC improve and plateau smoothly, reflecting stable convergence. These observations validate the effectiveness of the multi-objective optimization strategy adopted in the framework.

## 5 Discussion

The findings of this study highlight the novelty and effectiveness of the proposed deep learning-driven framework for the dynamic discovery and classification of virtualized intelligent transportation resources using API enrichment. Unlike traditional approaches that rely on either handcrafted features or isolated machine learning pipelines, the proposed design integrates spatiotemporal modeling, graph convolutional structures, transformer encoders, and enriched API features into a unified architecture. This combination enables the system to capture contextual dependencies across diverse data modalities, leading to improved accuracy, robustness, and anomaly detection capabilities. The novelty of this work lies not only in the multi-component integration but also in the adaptive synergy of discovery and classification tasks, where the joint optimization of reconstruction-based discovery and classification objectives contributes to more reliable intelligent transportation resource management.

Although the individual components of the proposed framework, including BiLSTM, Transformer encoders, graph convolutional networks, and autoencoder-based anomaly discovery, have been studied independently in prior intelligent transportation research, the novelty of this work lies in their unified integration within an API-enriched, multi-objective learning pipeline. Unlike existing hybrid ITS models that primarily focus on prediction or classification tasks, the proposed framework jointly performs contextual feature fusion, dynamic resource discovery, and supervised classification within a single optimization process. This design enables adaptive identification of transportation resources under evolving traffic conditions while leveraging external contextual signals, thereby extending beyond conventional spatiotemporal or graph-based modeling approaches that treat these objectives separately.

Compared with existing GNN-based traffic forecasting and Transformer-driven temporal modeling approaches, the proposed framework introduces several design distinctions. First, rather than optimizing a single predictive objective, the model jointly integrates anomaly discovery and supervised classification within a unified multi-objective learning formulation. Second, API-driven contextual enrichment is incorporated directly into representation learning, enabling adaptive responses to external environmental factors that are typically handled as auxiliary inputs or ignored in prior work. Third, the integration of graph reasoning with sequence encoders is designed to support dynamic resource discovery rather than only traffic prediction, expanding the application scope toward adaptive transportation resource analytics. These design choices collectively contribute to improved robustness and consistent performance gains across heterogeneous datasets, demonstrating advancement beyond conventional hybrid spatiotemporal ITS models that focus primarily on forecasting accuracy alone.

While the proposed framework employs a concatenation-based API fusion followed by a dense transformation (Eq. 17), this design was intentionally selected to balance modeling effectiveness and computational efficiency. Although more sophisticated fusion strategies such as cross-attention or gated fusion could model complex interactions between intrinsic and API-derived features, the chosen approach provided stable performance while avoiding additional parameter overhead and training instability. The dense projection layer acts as a learned filtering mechanism that mitigates noise and heterogeneity in external API signals while preserving useful contextual information. Future work will investigate adaptive attention-based fusion mechanisms to further enhance robustness under highly dynamic conditions.

The ablation results also indicate that removing the Transformer encoder leads to only a modest reduction in accuracy (approximately 1.5%), suggesting that the BiLSTM captures most short- and medium-range temporal dependencies present in the datasets. However, the Transformer provides complementary long-range temporal context through self-attention, which improves representation stability and robustness within the joint discovery–classification framework. Although attention mechanisms introduce additional computational cost, the latency analysis demonstrates that the overhead remains within practical limits for real-time intelligent transportation deployments.

The implications of the results are significant for the domain of intelligent transportation systems and smart mobility. By demonstrating consistent improvements across multiple datasets—including accident and traffic volume data as well as their combined form—the proposed framework proves its capacity to generalize across heterogeneous sources of transportation information. The integration of enriched APIs allows the system to adapt in real time, thereby extending its practical applicability to scenarios such as dynamic resource allocation for autonomous vehicles, proactive congestion management, and predictive traffic safety monitoring. The superior performance in both classification and anomaly detection tasks indicates that the proposed framework can serve as a foundation for building resilient ITS infrastructures where reliability, adaptability, and efficiency are paramount. These findings also suggest potential for integration into city-wide digital twins, cooperative vehicular networks, and next-generation traffic control systems.

### Limitations and Threats to Validity

Despite these promising outcomes, several limitations and potential threats to validity of the present work must be acknowledged. First, while the proposed framework achieves high accuracy and robustness, the reliance on deep architectures inevitably introduces computational overhead, which may pose challenges for deployment in resource-constrained environments such as edge devices or low-power vehicular units. Although the latency analysis indicates acceptable inference times, further optimization is required to ensure scalability in real-world, large-scale deployments. While the presented ablation study evaluates the contribution of major architectural components, a comprehensive sensitivity analysis across all hyperparameters was beyond the scope of this work. During experimentation, training stability was assessed through repeated validation monitoring and consistent convergence behavior across runs, as reflected in the reported training dynamics. Nevertheless, future work will investigate systematic hyperparameter sensitivity and multi-run statistical evaluation to further quantify robustness. In addition, the computational complexity of the framework is primarily influenced by sequence encoding and graph propagation operations, which scale with sequence length and graph size. Although the current architecture maintains practical inference latency, training cost increases with larger transportation networks, motivating future exploration of model compression, efficient attention mechanisms, and distributed training strategies to improve scalability. Although the reported results demonstrate consistent performance improvements across datasets and evaluation metrics, extensive repeated-run experimentation and formal statistical significance testing were not included in the current study. During development, model stability was monitored through consistent convergence trends and validation behavior across training runs; however, future work will incorporate multi-run evaluation with variance reporting and statistical hypothesis testing to further quantify robustness and reproducibility under varying initialization and training conditions. Second, the current evaluation is restricted to two publicly available datasets, which, while diverse, may not fully capture the complexity and variability of global traffic patterns, multimodal transportation infrastructures, or adversarial conditions such as sensor failures and malicious data injections. Finally, while the enriched API features enhance contextual understanding, their availability and reliability may vary depending on the deployment environment, introducing practical challenges for standardization and interoperability. The use of two datasets representing specific geographic regions may introduce dataset-dependent learning behavior, potentially limiting generalization to unseen cities or traffic environments. Although the selected datasets provide complementary characteristics spanning urban accident records and highway traffic dynamics, broader validation across additional regions and sensing infrastructures would further strengthen robustness assessment. Future work will therefore evaluate the proposed framework on larger multi-city and cross-domain transportation datasets to better analyze generalization capability and reduce potential dataset-specific bias.

These limitations open several avenues for future work. One promising direction is the incorporation of federated and distributed learning paradigms to reduce communication bottlenecks and enhance privacy-preserving model training across decentralized ITS nodes. Further exploration of lightweight architectures and model compression techniques, such as knowledge distillation and quantization, can improve computational efficiency and support real-time inference on edge devices. Another direction is the extension of the framework to multimodal datasets that include not only accident and traffic volume records but also sensor feeds, connected vehicle data, and environmental factors such as weather and road conditions. Additionally, robustness against adversarial attacks and resilience to missing or corrupted API data remain open research challenges. Finally, the development of explainable AI mechanisms tailored for transportation contexts will be crucial to ensure transparency, trust, and adoption in safety-critical domains.

In summary, the discussion underscores the novelty of integrating enriched APIs with deep spatiotemporal learning for intelligent transportation resource discovery and classification, highlights the strong implications for real-world smart mobility systems, acknowledges current limitations, and points toward concrete research directions for extending and refining the framework in future work.

## 6 Conclusions

This paper proposed a novel deep learning-driven framework for the dynamic discovery and classification of virtualized intelligent transportation resources using API enrichment, addressing critical challenges in intelligent transportation systems. By unifying spatiotemporal deep feature extraction, graph convolutional modeling, transformer-based sequence learning, and enriched API-driven context, the framework achieved superior performance across both classification and anomaly detection tasks. Experimental evaluations on the Barcelona Accident dataset, the Metro Interstate Traffic Volume dataset, and their combined form consistently demonstrated that the proposed approach outperforms traditional machine learning models and competitive deep learning baselines. The results confirm that enriched APIs play a crucial role in enabling real-time adaptability and enhancing predictive accuracy, while the integration of discovery and classification objectives fosters robustness and reliability in dynamic ITS environments. Moreover, the ablation study highlighted the contribution of each architectural component, validating the necessity of their synergistic integration. Beyond accuracy, the framework was shown to maintain acceptable inference latency, underscoring its practical feasibility for real-world deployments. While the proposed framework demonstrates consistent improvements across the evaluated datasets, the conclusions should be interpreted within the scope of the experimental setting. The study relies on two publicly available datasets representing specific traffic environments, and therefore broader generalization to different geographic regions, traffic infrastructures, or sensing conditions requires further validation. In addition, although regularization and validation monitoring were employed to mitigate overfitting, deep learning models may exhibit dataset-specific adaptation when trained on limited domain distributions. Future investigations involving larger-scale, multi-region datasets and diverse operational conditions will be necessary to fully establish the general applicability of the proposed approach. While certain limitations remain, such as computational overhead and dataset scope, the proposed method establishes a strong foundation for future extensions involving federated learning, multimodal data integration, lightweight model design, and resilience to adversarial conditions. Overall, the study contributes a significant step forward in advancing resilient, scalable, and adaptive ITS solutions, with the potential to support next-generation smart city infrastructures and autonomous mobility systems.
